# Factors associated with long-acting and short-acting reversible contraceptives use among 10–24-years-old youths in Lilongwe, Malawi

**DOI:** 10.3389/frph.2022.949458

**Published:** 2022-09-20

**Authors:** George Baxton Maruwo, Wingston Felix Ng'ambi, Adamson Sinjani Muula, Khumbo Zonda, Fannie Kachale

**Affiliations:** ^1^Management Sciences for Health, Lilongwe, Malawi; ^2^Department of Public Health, College of Medicine, Kamuzu University of Health Sciences, Blantyre, Malawi; ^3^Health Economics and Policy Unit, College of Medicine, Kamuzu University of Health Sciences, Lilongwe, Malawi; ^4^Global Research and Analyses for Public Health (GRAPH) Network, University of Geneva, Geneva, Switzerland; ^5^Institute of Global Health, University of Geneva, Geneva, Switzerland; ^6^Banja La Mtsogolo, Lilongwe, Malawi; ^7^Ministry of Health, Reproductive Health Directorate, Lilongwe, Malawi

**Keywords:** contraceptives, youth, LARC, SARC, reversible contraceptives, Lilongwe, Malawi

## Abstract

**Background:**

Evidence suggests that Malawi continues to register substantial progress on key Family Planning (FP) indicators. However, FP adoption is still low among married youth (15–24 years old), only 38% of married girls use modern contraceptive methods coupled with high-unmet needs (22%) in the same age group.

**Objective:**

Identifying factors associated with long-acting (LARC) and short-acting (SARC) reversible contraceptive use among 10–24-years-old youth in Lilongwe, Malawi.

**Methodology:**

A retrospective study using secondary data from 64 youth outreach clinic sites in the Lilongwe district. A quantitative approach using secondary data that was analyzed in STATA version 14 was used. A sample of 14,954 youth who accessed FP and Reproductive Health (RH) services during youth outreach clinics were included in the study.

**Results:**

SARC uptake was higher than LARC (*p* < 0.01). Of the youths who accessed FP methods, LARC uptake was 25% (*n* = 3,735). Variations were noted in the uptake of LARC, especially on age, education level, client status occupation, and marital status. Factors associated with LARC uptake varied; new clients were almost twice likely to use LARC (AOR = 1.87, CI: 1.59–2.19, *P* < 0.01) while youth aged 20–24, the single, and student youth were less likely to use LARC. Compared to young women with formal occupations, students were less likely to use LARC (AOR = 0.30, CI: 0.158–0.58, *P* < 0.01). Related to the number of living children, youths with a living child were likely to use LARC (AOR = 6.40, CI: 3.91–10.48, *P* < 0.01).

**Conclusion:**

This study showed that LARC uptake in youth outreach clinics in Lilongwe is low, though increasing over time. In addition to this, this study shows that SARC uptake is high among youth compared to LARC. Furthermore, LARC uptake varied by age education, client status (new, existing, and unknown client), occupation, number of living children, and marital status, and there were variations in LARC uptake by the clinic. Current outreach services reach youth with LARC services, but gaps exist for underserved youths.

## Introduction

### Background

Malawi's population (18 million people) is almost 4 times that of 1966 and 1.3 times of the 2008 census; so far the total population has increased by 35% from 2008 to 2018 (1). With an annual population growth rate of 2.9%, Malawi's population is forecast to double and reach 35 million by 2042 (1). The fertility rate of five births per 1,000 women is still high. Despite a contraceptive prevalence rate of 60% for married women, only 38% of married youth (15–19 years old) currently use a modern contraceptive method, and similarly, data for unmarried sexually active youth (15–24 years old) show that only 37% currently use a modern FP method. Malawi continues to face challenges with a total fertility rate of 5 children per woman and an unmet need for Family Planning of 19.4% (2). The high fertility rate and unmet needs bring about an increase in population growth, which outpaces economic growth and puts pressure on all sectors, health being the most affected. This calls for efforts to help families to achieve their reproductive goals, such as access to FP services, girls' education, and child mortality (3). With a very young population, two-thirds under the age of 24, it is especially crucial to consider how to reach youth with modern methods of contraception, particularly long-acting reversible methods, such as implants and Intra Uterine Devices (IUD).

### Policy environment

Over the years, Malawi had introduced policies to assist increase access to SRH services for adolescents. A review of Adolescent FP policies in Malawi in 2017 found that the policy environment is highly supportive of FP use by adolescents scoring 92.4% out of possible 100 points (4). Despite this favorable policy environment, youths still shun facility-based Youth Friendly Health Services (YFHS) citing doubts about their privacy and confidentiality. Other youths interviewed pointed out that they avoid YFHS because some providers require youth to undergo a HIV test before other health services are offered. While others cited barriers in access due to inadequate YFHS within the communities where a majority of the youth live (5). Only 1 in 3 unmarried girls and 2 in 5 boys are currently using a modern method of contraception. The average Malawian girl will have her first sexual encounter at age 17, be married by age 18, and have her first child by age 19 (2). Furthermore, family planning adoption is low among married adolescents (15–19 years old); only 38% of married girls in this age group currently utilize a modern contraceptive method. Similarly, data for unmarried sexually active women (15–24) shows that 37% currently use a modern method of FP (6). Short-term methods are most popular among teens: 9.1% use injectables, 3.7% use male condoms, and 1.7% use implants. Even if more youth use injectables, 41% of those 15–19 years discontinue within the first year of use. An evaluation of the YFHS in 2014 found that 37.1% of adolescent girls had heard of YFHS where integrated Sexual Reproductive Health (SRH) services, including contraception, are provided and that only 13% had used them. A clear gap exists in the use of contraception, especially with short-acting reversible contraceptives (SARC) vs. long-acting reversible contraceptives (LARC) (7). In this study, LARCs include Implanon, Jadelle, Levoplant, and Copper IUDs, while SARCs include pills, condoms, and injectables. Increasing access to contraception for youth has several benefits, including reduced rates of unintended pregnancies, unsafe abortions, and sexually transmitted infections. Moreover, delayed pregnancies lead to improved maternal and child health across the population, while allowing youth and adolescents to continue with their education, develop psychosocial skills, better develop their human capital, and contribute to the development of their communities and Malawi as a whole. Unfortunately, youth and adolescents often face obstacles in accessing contraceptives and health services, which increases the risk of unintended pregnancies (8).

## Methods

### Study design and sampling strategy

This is a retrospective study using secondary data analysis of clients accessing FP services from 64 youth outreach sites in Lilongwe districts. A total of 25,819 clients were registered on the database, and the second study used all records of 14,954 young people who accessed services within the youth outreach sites. With the available number of clients, the study has 80% power, 95% confidence interval, and 5% margin of error to detect a minimum difference of 30% for explanatory variables between those accessing SARCs and LARCs. The United Nations (UN) define youth as those people aged between the ages of 15–24 (UN) (9). However, in other studies, people aged 10–24 years are also considered, and the latter definition is used in this study (10).

### Study area and population

The study was conducted in 64 outreach sites, emanating from 10 out of the 19 traditional authorities, 18 Health Facilities (one hospital) from a population of 1,348,723 people in Lilongwe Urban (three facilities) and Rural (15 facilities) representing 51% of the district population. Lilongwe district has a population of 2,626,901 people. Out of these, 922,837 (35.1%) are youths of 10–24 years of age (1). The study focused on 25,819 clients attending services at the youth outreach clinics and the sub-analysis focused on 14,954 young people aged 10–24 years who accessed long-acting and short-acting reversible contraceptives within youth outreach clinics. Clients outside the 10–24 age bracket of young people (11) and those with missing variables, e.g., age, education, location, marital status, and living children, were excluded from the study. This is because the age groups were outside the WHO definition of young people, which is the primary focus of the study and the youth outreach program (11).

### Data collection and analysis

The primary data were collected using a structured questionnaire customized to draw information from the health records of clients between January 2018 and December 2019. The client record forms were collected during FP service provision in the youth outreach clinics. Data was then entered on a laptop into the Client Information Center (CLIC) form used by Banja La Mtsogolo. There were two data collection points, i.e., at client registration and the point of exit from the clinic site on the same day. Project drivers were trained by BLM to collect and enter data on laptops. For this secondary study, the investigator used the following data collected: demographic information (for example, age, education, occupation, and residence), clinical information (e.g., marital status, client status, and living children), and services provided (FP counseling and contraceptives provided).

Primary data were exported to Microsoft Excel 2016 from the Client Information Center (CLIC). Data cleaning for this second study was done in Microsoft Excel 2016 since not all the data was cleaned at the point of collection. Codes that were used in CLIC were maintained. Necessary coding was done as follows: questions with binary outcomes of yes or no were coded as 1 or 0, respectively. Thereafter, the data was imported into STATA version 14.1 for statistical analysis. A descriptive analysis including frequencies and proportions was calculated. Associations were determined as follows: initially, a bi-variate analysis was performed followed by a multi-variate (Logistic regression) analysis controlling for confounding effects and clustering by the clinic. The results were statistically significant if the probability (*p*-value) of finding the observed value was <0.05. Variables, which showed a significant association in the bivariate analysis, were further analyzed in the multi-variate analysis to look for a statistical association between LARCs and SARCs use. Aside from this, a further analysis was done to identify factors that were a true association with use of LARCs and SARCs while controlling for confounders.

### Ethical consideration

Ethical clearance (P.09/20/3139) was secured from the College of Medicine and Research Ethics Committee (COMREC), and permission to conduct the study was obtained from Banja La Mtsogolo. Informed consent was administered initially when collecting data for contact messages. This was obtained using client record forms that were clearly marked, “strictly confidential.” Confidentiality was maintained during the entire period of the study, only the Principal Investigator, Research Analysts, and Research statistician had access to the data, and it was stored on Dropbox to ensure that its contents are secured and protected from tampering. In addition to this, client identification and no names were used to ensure participants' confidentiality.

## Results

### Characteristics of youth included in the study

Table 1 summarizes the characteristics of youths in the study. A total of 14,954 youths were included in the study. Of these, many (62% *n* = 9,265) were aged between 20 and 24 years, while 36% (*n* = 5,306) were aged 15–19 years, with 1% (*n* = 86) aged 10–14 years. Regarding client status, 91% (*n* = 13,588) were new clients, while 9% (*n* = 1,336) were exiting clients, and 0.2% (*n* = 30) were unknown clients who had been accessing services from previous years. Only 2% (*n* = 306) had no formal education, while 82% (*n* = 12,304) had a primary school education level of education. Almost 75% (*n* = 11,219) were living in rural areas, while 25% (3,735) were from urban areas. Regarding the number of living children, 39% (*n* = 5,791) had zero children, 35% (*n* = 5,216) had one child living, and 19% (*n* = 2,872) had two living children, while 7% (*n* = 1,075) had three plus number of living children.

**Table 1:** Characteristics of youth who assessed LARC in youth outreach clinics in Lilongwe between 2018 and 2019.

**Characteristics**	**Total**	
	**Number (*n*)**	**%**
**Total**	14,954	100.0
**Age group**		
10–14	86	0.6
15–19	5,306	35.5
20–24	9,265	62.0
**Sex**		
Female	9,304	62.2
Male	5,650	37.8
**Education level**		
None	306	2.0
Primary	12,304	82.3
Secondary	1,939	13.0
Tertiary	405	2.7
**Client status**		
New client	13,588	90.9
Existing client	1,336	8.9
Unknown	30	0.2
**Residence**		
Urban	3,735	25.0
Rural	11,219	75.0
**Marital status**		
Divorced	123	0.8
Married	9,088	60.8
Single	5,743	38.4
**Occupation**		
Formal	147	1.0
Informal	2,686	18.0
Student	3,036	20.3
Unemployment	5,350	35.8
**Number of living children**		
0	5,791	38.7
1	5,216	34.9
2	2,872	19.2
3+	1,075	7.2

n, number; %, percentage.

### Trends in the uptake of SARCs and LARCs

Of the 14,954 youths that accessed services within the youth outreach clinics in Lilongwe, 25% (*n* = 3,735) of them accessed LARC within the 2018 and 2019 periods. SARCs were consistently higher than LARCs over the study period (*p* < 0.01). By September 2018, there was a monthly decreasing trend of SARCs while there was an increasing trend of LARCs. Between September 2018 and March 2019, there was some cycling regarding LARC and SARC uptake, whereas after March 2019 there was a decreasing trend in LARC and an increasing trend in SARC.

### Access to SARC and LARC by characteristics of the youths

The characteristics of the youth included in the study are shown in Table 2. A total of 14,954 youth accessed SARC and LARC services. Of these, 75% (*n* = 11,219) accessed SARCs and 25% (*n* = 3,735) accessed LARCs. The majority of the new clients assessed SARCs (74%, *n* = 10,097) while 26% (*n* = 3,491) of them accessed LARCs, and a similar picture is seen in existing clients with 82% (*n* = 109) accessing SARCs and 18% (*n* = 243) accessing LARCs (*P* < 0.01). There were more tertiary education youths who accessed SARCs (98%, *n* = 396) than those who accessed LARCs (2%, *n* = 9) within the youth outreach clinics. The majority of youths who reside in urban areas accessed SARCs (84%, *n* = 384) and only 16% (*n* = 71) accessed LARC. This is similar to those residing in rural areas with 75% (*n* = 10,835) accessing SARCs and 25% (*n* = 3,664) assessed LARCs (*P*-value < 0.01). The majority of single youths accessed SARCs (99%, *n* = 5,676) while only 1% (*n* = 67) accessed LARCs, and more married youths (60%, *n* = 5,461) assessed SARCs compared to 40% (*n* = 3,627) who accessed LARCs. There were more youths who were not employed who assessed SARCs (64%, *n* = 5,350) than those who assessed LARCs (36%, *n* = 2,962), and the majority of youths, who were students, were more likely to use SARCs (99%, *n* = 3,030) while only a few (0.8%, *n* = 25) assessed LARC services (*P*-value < 0.01). Regarding the number of living children, there were more youths with no living child (99%, *n* = 5,756) who accessed SARCs compared to those who assessed LARC (1%, *n* = 35) and the use of LARC was more prominent among the youths with 2 or more living children (>40%, *n* = 1,261 and *n* = 443, respectively).

**Table 2:** Proportion of persons aged 10–24 years that accessed SARC and LARC in Lilongwe outreach clinics between 2018 and 2019.

**Characteristics (n=14,954)**	**Total**		**SARC**		**LARC**		***P*-value**
	***n***	**%**	***n***	**%**	***n***	**%**	
**Total**	14,954	100.0	11,219	75.0	3,735	25.0	*P* < 0.01
**Age group**							
10–14	86	100.0	86	100.0	0	0.0	*P* < 0.01
15–19	5,306	100.0	4,526	80.8	1,077	19.2	
20–24	9,265	100.0	6,607	71.3	2,658	28.7	
**Sex**							
Female	9,304	100.0	5,569	59.9	3,735	40.1	*P* < 0.01
Male	5,650	100.0	5,650	100.0	0	0.0	
**Education level**							
None	306	100.0	204	66.7	102	33.3	*P* < 0.01
Primary	12,304	100.0	8,895	72.3	3,409	27.7	
Secondary	1,939	100.0	1,742	88.9	215	11.1	
Tertiary	405	100.0	396	97.8	9	2.2	
**Client status**							
New client	13,588	100.0	10,097	74.3	3,491	25.7	*P* < 0.01
Existing client	1,336	100.0	1,093	81.8	243	18.2	
Unknown	30	100.0	29	96.7	1	3.3	
**Residence**							
Urban	3,735	100.0	384	84.4	71	15.6	*P* < 0.01
Rural	11,219	100.0	10,835	74.7	3,664	25.3	
**Marital status**							
Divorced	123	100.0	82	66.7	41	33.3	*P* < 0.01
Married	9,088	100.0	5,461	60.1	3,627	39.9	
Single	5,743	100.0	5,676	98.8	67	1.2	
**Occupation**							
Formal	194	100.0	147	75.8	47	24.2	*P* < 0.01
Informal	2,686	100.0	2,686	79.3	700	20.7	
Student	3,055	100.0	3,030	99.2	25	0.8	
Unemployment	5,350	100.0	5,350	64.4	2,962	35.6	
**Number of living children**							
0	5,791	100.0	5,756	99.4	35	0.6	*P* < 0.01
1	5,216	100.0	3,220	61.7	1,996	38.3	
2	2,872	100.0	1,611	56.1	1,261	43.9	
3+	1,075	100.0	632	58.8	443	41.2	

SARC, Short-acting reversible contraceptives; LARC, long-acting reversible contraceptives.

### Factors associated with LARC uptake

The factors associated with LARC uptake are shown in Table 3, LARC uptake varied by age, education, client status, occupation, and marital status. Those aged 20–24 years were less likely to use LARC (AOR = 0.52, CI: 0.46–0.58, *P* < 0.01). After controlling for age, client status, occupation and marital status, and an increase in education level were associated with a decrease in the trend of LARC uptake although there was weak evidence of an association between LARC uptake and education level. Compared to the divorced women, the singles were less likely to use LARC (AOR = 0.62, CI: 0.35–1.10, *P* < 0.10). After controlling for age, education, occupation, and marital status, the new clients were almost twice likely to access LARC (AOR = 1.83, CI: 1.56–2.16, *P* < 0.01). Compared to women with formal occupation, student women were less likely to use LARC (AOR = 0.57, CI: 0.28–1.13, *P* < 0.11). Regarding the number of living children, youths with a living child were likely to use LARC (AOR = 6.40, CI: 3.91–10.48, *P* < 0.01). The crude and adjusted OR vary by an average of 21% and this shows that the crude OR may not better demonstrate the effect of LARC uptake, hence the use of AOR is a better choice for assessing the effect of LARC uptake in this population between 2018 and 2019.

**Table 3:** Bivariate and multivariate odds ratios for factors associated with long-acting reversible contraceptives (LARC) use among 10–24-years-old youths in Lilongwe.

**Characteristics (*n* = 14,954)**	**Bivariate analysis**		**Multivariate analysis**	
	**OR (95%CI)**		**OR (95%CI)**	
**Age group**				
15–19	1		1	
20–24	0.74 (0.67–0.82)	<0.00	0.52 (0.46 −0.58)	<0.01
**Education level**				
None	1		1	
Primary	0.83 (0.63–1.09)	0.17	0.77 (0.59–1.02)	0.07
Secondary	0.66 (0.48–0.91)	0.01	0.84 (0.60–1.17)	0.3
Tertiary	0.14 (0.06–0.36)	<0.01	0.81 (0.32–2.04)	0.65
**Client status**				
Existing	1		1	
New client	1.96 (1.67–2.29)	<0.01	1.83(1.56–2.16)	<0.01
Unknown	3.18 (1.00–51.59)	0.42	2.44 (0.15–39.83)	0.53
**Residence**				
Urban	1			
Rural	0.41 (0.19–0.91)	0.03		
**Marital status**				
Divorced	1		1	
Married	0.97 (0.64–1.47)	0.09	0.88 (0.58–1.34)	0.56
Single	0.20 (0.12–0.33)	<0.01	0.62 (0.35–1.10)	<0.10
**Occupation**				
Formal	1.00		1.00	
Informal	0.76 (0.52–1.12)	0.17	0.75 (0.50–1.12)	0.16
Student	0.13 (0.074–0.236)	<0.01	0.57 (0.28–1.13)	0.11
Unemployment	1.23 (0.84–1.80)	0.28	1.10 (0.74–1.62)	0.65
**Number of children ever born**				
0.00	1.00		1.00	
1	8.89 (6.15–12.8)	<0.01	6.40(3.91–10.48)	<0.01
2	10.7 (7.39–15.52)	<0.01	9.60 (5.81–15.85)	<0.01
3+	9.77 (6.65–14.35)	<0.01	9.04 (5.42–15.08)	<0.01

## Discussion

The aim of the study was to assess factors associated with long-acting and short-acting reversible contraceptives use among 10–24-years-old youth in Lilongwe. Hence, we investigated a total sample of 14,954 clients who accessed SARC and LARC services in youth outreach clinics in Lilongwe. This study specifically focused on factors associated with LARC and SARC use among youth accessing outreach clinics. This chapter, therefore, will discuss the study findings from the secondary data analysis.

**Figure 1:**
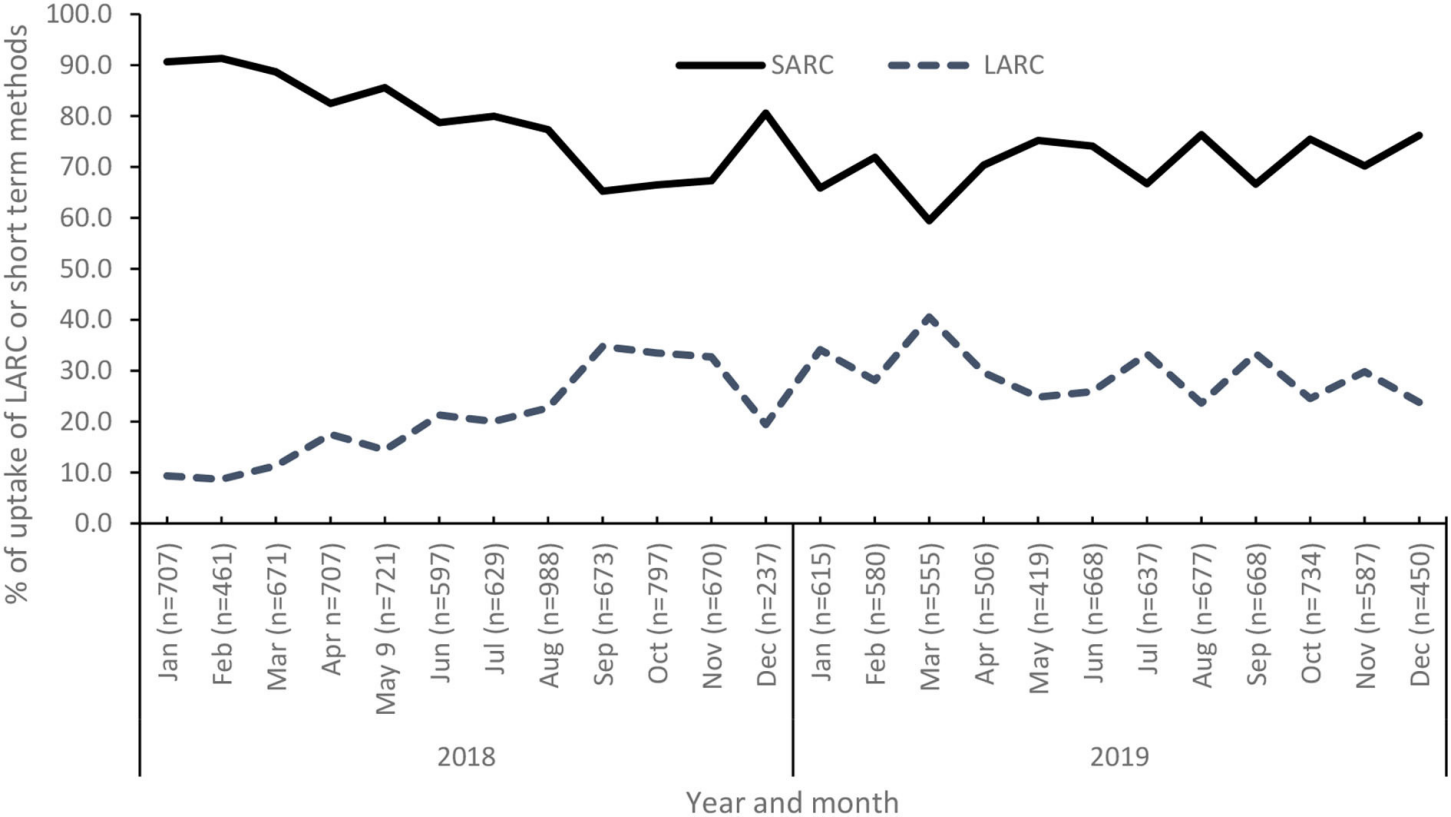
Trends in the uptake of SARC and LARC services among youth in Lilongwe between 2018 and 2019. SARC, Short-acting reversible contraceptives; LARC, long-acting reversible contraceptives.

In this study, the following were key findings as captured in Figure 1. LARC uptake was increasing monthly among youth who accessed FP services in the youth outreach clinics. This is an encouraging finding as it will contribute to the reduction of unintended pregnancies among the youth, and hence reduce unmet needs, and increase in contraceptive prevalence rate for the Lilongwe district. Studies have reported similar findings with a steady but slow upward trend in LARC methods use. This was noted across selected countries where a marginal increase was recorded in LARC uptake over a 10-years period in many of the chosen countries (12). Within the proportion of youth who accessed SARC and LARC services, we noted that the older the youth the less likely they will use LARC (*p* < 0.01). This is because most of the youths who access services within the outreach clinics were already married or in a union. Another study by Nyambo et al. on factors influencing uptake of LARC in Malawi noted similar findings, i.e., the study found that younger women between 15 and 24 years old are more likely to use LARC methods than older women (13). In most cases, women within this age group would have their first pregnancy and would not want another child within 3 years, hence, they opt for a long-lasting FP method; the Malawi Demographic and Health Survey reported similar findings (4). The higher unmet need within this age group also highlights the need to target interventions for first-time parents (14).

Another study done by Marie Stopes International evaluated the effectiveness of a similar program aimed at expanding access to a range of LARC methods and addressing the unmet need in 11 Sub-Sahara African countries between 2008 and 2012. Many youths have a high-unmet need for contraception, and reaching them with services within the communities where they live can help to improve method choice. In another study done for countries in Sub-Saharan Africa, 42% of the FP clients who accessed services at outreach sites were living on less than a dollar per day and hence would not access services if the services are brought closer to them (15). Therefore, the youth outreach clinics serve a niche of unique clients as far as LARC access is concerned. The previous finding is consistent with what was noted in this study; uptake of LARC was at 25% among youth accessing LARC services in Lilongwe. This indicates good progress in youths accessing LARC services, the Malawi Demographic Health Survey 2015–2016 reports that only 13.3% of youths use LARC, and the percentage drops to 1.7% for adolescents, yet these age groups have a high-unmet need for contraception and are the least to use FP methods (4).

Nyambo et al. in a study conducted in 2013 reported a LARC prevalence of 26% and concluded that with well-crafted strategies, young people are more likely to use LARC methods than older women in Malawi (16). We also found that SARCs' use was consistently higher than LARCs' use among the youth over the study period. This is consistent with other studies on SARC uptake by the youths, which reported that 47.5% of youth use short-term methods of contraception. It is worth noting that SARCs' use is common among youth but with high discontinuation rates compared to LARC (2). The Malawi demographic and health survey of 2015–2016 reported that injectables accounted for 41% of discontinuation rates, 61.9% for Pills and Condoms, and only 7.6% for LARC among 15–24-years-old young women (4). This is due to a number of factors, which include barriers to access to other options of contraception as most LARC services are provided at the facility level, and at times erratic supply of LARC reduces access. Alternative LARC service delivery points, i.e., in private clinics are costly for youths (17).

Vested myths, misconceptions, and fear of side effects also account for the non-use of long-term methods coupled with gaps in the capacity of providers to perform implant insertion and removal, including IUCD. A study done by Self and co-workers in 2018 on drivers, barriers, and suggestions for youth access to contraception in Dowa, Machinga, and Phalombe alludes to the challenges in access to LARC (18). LARCs offer an opportunity to promote sustained use of contraception. The confusion on laws and policies regarding the age when adolescents can access SRH services needs to be tackled to ensure that the national commitments are addressing barriers and that they promote a model of greater reproductive autonomy for adolescents (19).

Remarkably, the study noted some variations in LARC's uptake by age, education, client status, occupation marital status, and the number of living children. It was noted that students, youths of 20–24 years, were less likely to use LARC, while new clients and the divorced were more likely to use LARC. LARC use across sites varied by 13%, and urban areas, i.e., Natural resources, Lilongwe Technical, and Bunda colleges recorded no LARC uptake. It was also noted that the number of living children was associated with the likelihood of using LARC. A study done by Silviana and co-workers in 2019 on the relation between the number of living children and long-acting and permanent contraceptive methods reported similar findings (14). Since the study only used secondary data, future studies might help to explore factors associated with low LARC uptake, especially in tertiary institutions and in youth outreach sites, as these were termed as safe spaces for youths to access FP/RH services (20).

## Limitations of the study

We used secondary data; therefore, the data on religion, fertility intentions, level of income, and provider views on LARC and SARC were not captured for use in the analysis. These data could provide more information on other factors associated with long-acting and short-acting reversible contraceptives uptake. Furthermore, as much as clinical information was available on the data collection tool, the organization's implementation of the program only released data without other clinical variables, i.e., Weight, Blood pressure measurements, parity, and HIV status. However, regarding the parity variable, the researcher used the number of living children as a proxy. Nevertheless, this study fills an important gap as far as literature on LARC use within youth outreach clinics in Lilongwe is concerned.

## Conclusion

The study suggests that long-acting reversible contraceptives (LARC) uptake in youth outreach clinics in Lilongwe is increasing over time. The study findings further indicate that short-acting reversible contraceptives (SARC) uptake is high among the youth compared to LARC. In addition to this, the study showed that LARC uptake varied by age education, client status, occupation marital status, and the number of living children. This study also noted variations in LARC uptake by clinics. Therefore, this study underscores for the need the existence of barriers in access to contraception, especially with LARC as opposed to SARCs among youth within outreach clinics. Another dimension on contraception choices is noted among in-school youths at both secondary and tertiary institutions with more preference to short-term contraceptives.

## Data availability statement

The raw data supporting the conclusions of this article will be made available by the authors, without undue reservation.

## Ethics statement

The studies involving human participants were reviewed and approved by College of Medicine, University of Malawi Research and Ethics Committee. Written informed consent from the participants' legal guardian/next of kin was not required to participate in this study in accordance with the national legislation and the institutional requirements.

## Author contributions

AM contributed to conception and design of the study. KZ organized the data from CLIC database. WN supported to execute the statistical analysis. AM and WN supported with writing of the manuscript sections. All authors contributed to manuscript revision, read, and approved the submitted version.

## Funding

This is a self-sponsored research paper using funds from the researcher earnings while working with Management Sciences for Health.

## Conflict of interest

Author GM was employed by Management Sciences for Health. Author KZ was employed by Banja La Mtsogolo. The remaining authors declare that the research was conducted in the absence of any commercial or financial relationships that could be construed as a potential conflict of interest.

## Publisher's note

All claims expressed in this article are solely those of the authors and do not necessarily represent those of their affiliated organizations, or those of the publisher, the editors and the reviewers. Any product that may be evaluated in this article, or claim that may be made by its manufacturer, is not guaranteed or endorsed by the publisher.
